# *N*-[3-(*tert*-Butyl­dimethyl­siloxymeth­yl)-5-nitro­phen­yl]acetamide

**DOI:** 10.1107/S1600536808030717

**Published:** 2008-09-27

**Authors:** Gul S. Khan, George R. Clark, David Barker

**Affiliations:** a Chemistry Department, University of Auckland, Private Bag 92019, Auckland, New Zealand

## Abstract

The title compound, C_15_H_24_N_2_O_4_Si, was prepared by the reaction of (3-acetamido-5-nitro­benz­yl)methanol with *tert*-butyl­dimethyl­silyl chloride and is a key inter­mediate in the synthesis of novel nonsymmetrical DNA minor groove-binding agents. There are two independent mol­ecules in the structure, which differ primarily in the rotation about the C—O bond next to the Si atom. Two strong N—H⋯O hydrogen bonds align the mol­ecules into a wide ribbon extending approximately parallel to the *b* axis.

## Related literature

For literature related to protecting groups, see: Jarowicki & Kocienski (1998 ▶); Kocienski (2004 ▶); Schelhaas & Waldmann (1996 ▶); Wetter & Oertle (1985 ▶); Wuts & Green (2006 ▶). For literature related to benzamides as minor groove binders, see: Barker *et al.* (2008 ▶); Gong & Yan (1997 ▶). For related literature, see: Crouch (2004 ▶); Desiraju & Steiner (1999 ▶); Nelson & Crouch (1996 ▶).
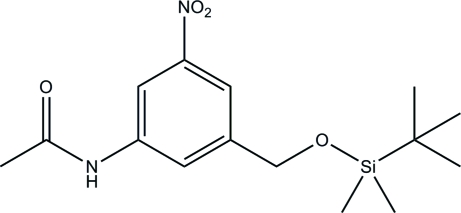


## Experimental

### 

#### Crystal data

C_15_H_24_N_2_O_4_Si
*M* *_r_* = 324.45
Triclinic, 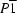

*a* = 9.5037 (3) Å
*b* = 10.0713 (3) Å
*c* = 18.1985 (5) Å
α = 89.885 (1)°
β = 86.009 (1)°
γ = 88.888 (1)°
*V* = 1737.31 (9) Å^3^
*Z* = 4
Mo *K*α radiation
μ = 0.15 mm^−1^
*T* = 90 (2) K
0.34 × 0.22 × 0.20 mm

#### Data collection

Siemens SMART CCD diffractometer
Absorption correction: multi-scan (*SADABS*; Sheldrick, 1997 ▶) *T*_min_ = 0.878, *T*_max_ = 0.977
15951 measured reflections
6570 independent reflections
4732 reflections with *I* > 2σ(*I*)
*R*_int_ = 0.042

#### Refinement

*R*[*F* ^2^ > 2σ(*F* ^2^)] = 0.048
*wR*(*F* ^2^) = 0.117
*S* = 1.05
6570 reflections
409 parameters
H-atom parameters constrained
Δρ_max_ = 0.41 e Å^−3^
Δρ_min_ = −0.31 e Å^−3^

### 

Data collection: *SMART* (Bruker, 1995 ▶); cell refinement: *SAINT* (Bruker, 1995 ▶); data reduction: *SAINT*; program(s) used to solve structure: *SHELXS97* (Sheldrick, 2008 ▶); program(s) used to refine structure: *SHELXL97* (Sheldrick, 2008 ▶); molecular graphics: *ORTEPIII* (Burnett & Johnson, 1996 ▶); software used to prepare material for publication: *SHELXTL* (Sheldrick, 2008 ▶).

## Supplementary Material

Crystal structure: contains datablocks I, global. DOI: 10.1107/S1600536808030717/fb2117sup1.cif

Structure factors: contains datablocks I. DOI: 10.1107/S1600536808030717/fb2117Isup2.hkl

Additional supplementary materials:  crystallographic information; 3D view; checkCIF report

## Figures and Tables

**Table 1:** Hydrogen-bond geometry (Å, °)

*D*—H⋯*A*	*D*—H	H⋯*A*	*D*⋯*A*	*D*—H⋯*A*
N1*A*—H1*A*⋯O1*B*	0.86	2.13	2.982 (2)	172
N1*B*—H1*B*⋯O1*A*^i^	0.86	2.14	2.991 (2)	173

Symmetry code: (i) 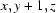.

**Fig. 1.**
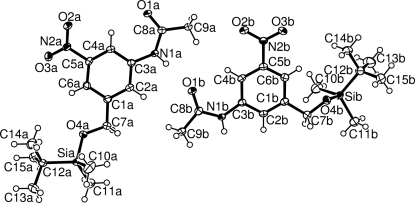
Structure showing 50% probability displacement ellipsoids for non-hydrogen atoms with hydrogen atoms as arbitary spheres (Burnett & Johnson, 1996). The two molecules differ mainly in the rotation about the C7 - O4 bond.

## supplementary crystallographic information

### Comment

Due to the nucleophilic nature of benzylic hydroxyl groups these are usually protected during multi-step organic synthesis (Barker *et al.*, 2008). Large numbers of protecting groups are reported including a variety of silyl ethers. Among the silyl ethers, the *tert*-butyldimethylsilyl ether is widely used due to it stability towards oxidative, reductive, and mild acidic and basic conditions (Jarowicki & Kocienski, 1998; Kocienski, 2004; Schelhaas & Waldmann, 1996; Wetter & Oertle, 1985; Wuts & Green, 2006). It can however be easily deprotected to give the parent hydroxyl group efficiently using different fluoride reagents without affecting other functionalities (Crouch, 2004; Nelson & Crouch, 1996). The asymmetric unit contains two independent molecules which differ primarily in the rotation about the C7 - O4 bond. (Torsion angles C1-C7-O4-Si equal to -152.75, -110.21° for molecules A and B respectively). Two strong N-H···O hydrogen bonds (Desiraju & Steiner, 1999; Tab. 1) align the molecules into wide ribbons extending approximately parallel to the *b* axis. There are four very close intramolecular contacts (C4A-O1A 2.886 (3), C4B-O1B 2.894 (3), C6A-O4A 2.740 (3) and C6B-O4B 2.785 Å).

### Experimental

To a solution of (3-acetamido-5-nitrobenzyl)methanol (alternatively 3-acetamido-5-nitrobenzyl alcohol) (150 mg, 0.714 mmol) in dry dimethylformamide (1 ml) under an atmosphere of nitrogen, was added *tert*-butyldimethylsilyl chloride (129 mg. 0.856 mmol) and imidazole (146 mg, 2.14 mmol) and the mixture stirred at room temperature, under an atmosphere of nitrogen for 2 h. Water (10 ml) was added, and the aqueous solution extracted with dichloromethane (2 *×* 10 ml). The combined organic extracts were washed with water (20 ml) and brine (20 ml), dried (MgSO_4_), filtered and the solvent removed *in vacuo* to afford the crude product, which was purified by flash chromatography (19:1 dichloromethane-methanol) to afford the title compound (214 mg, 93%) as a pale yellow solid, which was recrystallized from ethyl acetate and n-hexane to give a white crystal suitable for single-crystal analysis. (mp 416–417 K). ν_max_ (NaCl)/cm^-1^ 3315, 2929, 1666, 1532. δH (400 MHz, CDCl_3_) 0.13 (6*H*, s, OSi(C*H*_3_)_2_), 0.96 (9*H*, s, OSiC(C*H*_3_)_2_), 2.24 (3*H*, s, NHCOC*H*_3_), 4.79 (2*H*, s, ArC*H*_2_O), 7.56 (1*H*, s, NH), 7.91 (1*H*, s, Ar—H), 7.93 (1*H*, s, Ar—H) and 8.24 (1*H*, s, Ar—H). δC (100 MHz, CDCl_3_) -5.4 (CH_3_, OSi(*C*H_3_)_2_), 18.4 (quat. OSi*C*(CH_3_)_3_), 24.6 (CH_3_, NHCO*C*H_3_), 25.9 (CH_3_, OSiC(*C*H_3_)_3_), 63.8 (CH_2_, Ar*C*H_2_O), 112.9 (CH, Ar—C), 116.2 (CH, Ar—C), 122.4 (CH, Ar—C), 138.8 (quat. Ar—C), 144.6 (quat. Ar—C), 148.6 (quat. Ar—C) and 168.6 (C=O). *m/z* (CI^+^) 325 (MH^+^, 43%), 295 (*M*^+^—CH_2_O, 100) 267 (*M*^+^-NHCOCH_3_, 35), 221 (*M*^+^—C_2_H_4_N_2_O_3_, 32). Found MH^+^ 325.15862, C_15_H_25_N_2_O_4_Si requires 325.15836.

### Refinement

All the hydrogens were clearly discernible in the difference electron density map. Nevertheless, the hydrogens were placed in calculated positions and refined using the riding model under the conditions:. C_aryl_-H_aryl_=0.93, C_methyl_-H_methyl_=0.96, C_methylene_-H_methylene_ = 0.97, N-H = 0.86 Å, *U*_iso_(H) = 1.2*U*_eq_(C) except for the methyl hydrogens where *U*_iso_(H) = 1.5*U*_eq_(C).

### Crystal data

**Table:**

C_15_H_24_N_2_O_4_Si	*Z* = 4
*M**_r_* = 324.45	*F*(000) = 696
Triclinic, *P*1	*D*_x_ = 1.240 Mg m^−^^3^
Hall symbol: -P 1	Mo *K*α radiation, λ = 0.71073 Å
*a* = 9.5037 (3) Å	Cell parameters from 7491 reflections
*b* = 10.0713 (3) Å	θ = 2.0–25.7°
*c* = 18.1985 (5) Å	µ = 0.15 mm^−^^1^
α = 89.885 (1)°	*T* = 90 K
β = 86.009 (1)°	Plate, colourless
γ = 88.888 (1)°	0.34 × 0.22 × 0.20 mm
*V* = 1737.31 (9) Å^3^	

### Data collection

**Table:**

Siemens SMART CCD diffractometer	6570 independent reflections
Radiation source: fine-focus sealed tube	4732 reflections with *I* > 2σ(*I*)
graphite	*R*_int_ = 0.042
ω scans	θ_max_ = 25.7°, θ_min_ = 2.0°
Absorption correction: multi-scan (SADABS; Sheldrick, 1997)	*h* = −11→11
*T*_min_ = 0.878, *T*_max_ = 0.977	*k* = −12→12
15951 measured reflections	*l* = −22→22

### Refinement

**Table:**

Refinement on *F*^2^	Primary atom site location: structure-invariant direct methods
Least-squares matrix: full	Secondary atom site location: difference Fourier map
*R*[*F*^2^ > 2σ(*F*^2^)] = 0.048	Hydrogen site location: difference Fourier map
*wR*(*F*^2^) = 0.117	H-atom parameters constrained
*S* = 1.05	*w* = 1/[σ^2^(*F*_o_^2^) + (0.0481*P*)^2^ + 0.5796*P*] where *P* = (*F*_o_^2^ + 2*F*_c_^2^)/3
6570 reflections	(Δ/σ)_max_ = 0.001
409 parameters	Δρ_max_ = 0.41 e Å^−^^3^
0 restraints	Δρ_min_ = −0.31 e Å^−^^3^
180 constraints	

### Special details

**Table:**

Geometry. All e.s.d.'s (except the e.s.d. in the dihedral angle between two l.s. planes) are estimated using the full covariance matrix. The cell e.s.d.'s are taken into account individually in the estimation of e.s.d.'s in distances, angles and torsion angles; correlations between e.s.d.'s in cell parameters are only used when they are defined by crystal symmetry. An approximate (isotropic) treatment of cell e.s.d.'s is used for estimating e.s.d.'s involving l.s. planes.
Refinement. Refinement of *F*^2^ against ALL reflections. The weighted *R*-factor *wR* and goodness of fit *S* are based on *F*^2^, conventional *R*-factors *R* are based on *F*, with *F* set to zero for negative *F*^2^. The threshold expression of *F*^2^ > σ(*F*^2^) is used only for calculating *R*-factors(gt) *etc*. and is not relevant to the choice of reflections for refinement. *R*-factors based on *F*^2^ are statistically about twice as large as those based on *F*, and *R*- factors based on ALL data will be even larger.

### Fractional atomic coordinates and isotropic or equivalent isotropic displacement parameters (Å^2^)

**Table:**

	*x*	*y*	*z*	*U*_iso_*/*U*_eq_	
SiA	−0.17682 (7)	0.55743 (6)	0.16477 (4)	0.02135 (16)	
O1A	0.24286 (16)	0.04298 (15)	0.50611 (8)	0.0226 (4)	
O2A	−0.05223 (17)	−0.11050 (15)	0.34529 (9)	0.0266 (4)	
O3A	−0.22213 (17)	−0.00550 (16)	0.29506 (9)	0.0277 (4)	
O4A	−0.16983 (18)	0.45572 (16)	0.23590 (9)	0.0289 (4)	
N1A	0.19067 (19)	0.25787 (18)	0.47613 (10)	0.0198 (4)	
H1A	0.2094	0.3387	0.4864	0.024*	
N2A	−0.1138 (2)	−0.00728 (18)	0.32807 (10)	0.0210 (4)	
C1A	−0.0477 (2)	0.3543 (2)	0.33345 (12)	0.0205 (5)	
C2A	0.0484 (2)	0.3574 (2)	0.38736 (12)	0.0201 (5)	
H2A	0.0839	0.4386	0.4008	0.024*	
C3A	0.0934 (2)	0.2417 (2)	0.42199 (12)	0.0182 (5)	
C4A	0.0406 (2)	0.1201 (2)	0.40215 (12)	0.0189 (5)	
H4A	0.0687	0.0415	0.4242	0.023*	
C5A	−0.0547 (2)	0.1205 (2)	0.34864 (12)	0.0186 (5)	
C6A	−0.1015 (2)	0.2331 (2)	0.31340 (12)	0.0207 (5)	
H6A	−0.1664	0.2280	0.2776	0.025*	
C7A	−0.0922 (3)	0.4822 (2)	0.29796 (14)	0.0293 (6)	
H7A1	−0.1500	0.5350	0.3334	0.035*	
H7A2	−0.0094	0.5328	0.2826	0.035*	
C8A	0.2593 (2)	0.1632 (2)	0.51459 (12)	0.0197 (5)	
C9A	0.3541 (2)	0.2174 (2)	0.56920 (13)	0.0234 (5)	
H9A1	0.3141	0.2013	0.6182	0.035*	
H9A2	0.3638	0.3112	0.5616	0.035*	
H9A3	0.4451	0.1744	0.5627	0.035*	
C10A	0.0040 (3)	0.5812 (3)	0.12128 (17)	0.0410 (7)	
H10A	0.0640	0.6130	0.1575	0.061*	
H10B	0.0004	0.6448	0.0821	0.061*	
H10C	0.0409	0.4980	0.1019	0.061*	
C11A	−0.2521 (3)	0.7202 (2)	0.19618 (15)	0.0353 (6)	
H11A	−0.3412	0.7073	0.2232	0.053*	
H11B	−0.2656	0.7758	0.1542	0.053*	
H11C	−0.1886	0.7619	0.2274	0.053*	
C12A	−0.2923 (2)	0.4702 (2)	0.10128 (12)	0.0223 (5)	
C13A	−0.3086 (3)	0.5563 (3)	0.03232 (14)	0.0376 (7)	
H13A	−0.2169	0.5763	0.0099	0.056*	
H13B	−0.3581	0.6374	0.0461	0.056*	
H13C	−0.3608	0.5088	−0.0021	0.056*	
C14A	−0.2269 (3)	0.3351 (2)	0.07771 (15)	0.0329 (6)	
H14A	−0.2887	0.2907	0.0467	0.049*	
H14B	−0.2136	0.2820	0.1206	0.049*	
H14C	−0.1375	0.3482	0.0511	0.049*	
C15A	−0.4387 (2)	0.4478 (3)	0.14056 (14)	0.0290 (6)	
H15A	−0.4971	0.4032	0.1078	0.043*	
H15B	−0.4815	0.5319	0.1547	0.043*	
H15C	−0.4287	0.3943	0.1837	0.043*	
SiB	0.66924 (7)	1.00767 (6)	0.83117 (4)	0.02233 (17)	
O1B	0.26630 (16)	0.54068 (15)	0.49672 (9)	0.0252 (4)	
O2B	0.54486 (18)	0.38508 (16)	0.66784 (9)	0.0296 (4)	
O3B	0.71294 (17)	0.48319 (16)	0.71949 (9)	0.0279 (4)	
O4B	0.71134 (16)	0.96086 (16)	0.74525 (9)	0.0245 (4)	
N1B	0.31186 (19)	0.75499 (18)	0.52717 (10)	0.0190 (4)	
H1B	0.2892	0.8359	0.5175	0.023*	
N2B	0.6080 (2)	0.48532 (19)	0.68401 (10)	0.0216 (4)	
C1B	0.5633 (2)	0.8507 (2)	0.66133 (13)	0.0209 (5)	
C2B	0.4649 (2)	0.8537 (2)	0.60912 (12)	0.0194 (5)	
H2B	0.4337	0.9355	0.5920	0.023*	
C3B	0.4107 (2)	0.7380 (2)	0.58106 (12)	0.0191 (5)	
C4B	0.4566 (2)	0.6150 (2)	0.60630 (12)	0.0197 (5)	
H4B	0.4221	0.5361	0.5889	0.024*	
C5B	0.5557 (2)	0.6154 (2)	0.65841 (13)	0.0205 (5)	
C6B	0.6107 (2)	0.7283 (2)	0.68713 (12)	0.0211 (5)	
H6B	0.6769	0.7230	0.7224	0.025*	
C7B	0.6209 (3)	0.9803 (2)	0.68756 (13)	0.0262 (6)	
H7B1	0.6722	1.0235	0.6466	0.031*	
H7B2	0.5428	1.0385	0.7045	0.031*	
C8B	0.2475 (2)	0.6608 (2)	0.48844 (13)	0.0206 (5)	
C9B	0.1529 (2)	0.7165 (2)	0.43247 (13)	0.0229 (5)	
H9B1	0.1964	0.7023	0.3839	0.034*	
H9B2	0.1381	0.8100	0.4408	0.034*	
H9B3	0.0639	0.6728	0.4371	0.034*	
C10B	0.4914 (3)	0.9443 (3)	0.86052 (17)	0.0406 (7)	
H10D	0.4242	0.9766	0.8274	0.061*	
H10E	0.4642	0.9746	0.9095	0.061*	
H10F	0.4940	0.8489	0.8597	0.061*	
C11B	0.6654 (3)	1.1913 (2)	0.83711 (16)	0.0387 (7)	
H11D	0.7545	1.2248	0.8179	0.058*	
H11E	0.6483	1.2178	0.8876	0.058*	
H11F	0.5916	1.2264	0.8089	0.058*	
C12B	0.8116 (3)	0.9314 (2)	0.88521 (13)	0.0264 (6)	
C13B	0.7726 (3)	0.9463 (3)	0.96821 (15)	0.0454 (8)	
H13D	0.8460	0.9072	0.9953	0.068*	
H13E	0.6856	0.9021	0.9807	0.068*	
H13F	0.7618	1.0388	0.9804	0.068*	
C14B	0.8295 (3)	0.7825 (2)	0.86699 (15)	0.0333 (6)	
H14D	0.8574	0.7719	0.8156	0.050*	
H14E	0.7417	0.7389	0.8782	0.050*	
H14F	0.9007	0.7438	0.8958	0.050*	
C15B	0.9528 (3)	0.9991 (3)	0.86574 (16)	0.0354 (6)	
H15D	1.0253	0.9569	0.8922	0.053*	
H15E	0.9448	1.0912	0.8791	0.053*	
H15F	0.9766	0.9914	0.8138	0.053*	

### Atomic displacement parameters (Å^2^)

**Table:**

	*U*^11^	*U*^22^	*U*^33^	*U*^12^	*U*^13^	*U*^23^
SiA	0.0258 (3)	0.0145 (3)	0.0240 (4)	−0.0004 (3)	−0.0037 (3)	0.0021 (3)
O1A	0.0285 (9)	0.0141 (8)	0.0259 (9)	−0.0001 (7)	−0.0061 (7)	0.0015 (7)
O2A	0.0325 (9)	0.0144 (8)	0.0336 (10)	0.0024 (7)	−0.0090 (8)	−0.0033 (7)
O3A	0.0272 (9)	0.0239 (9)	0.0335 (10)	−0.0010 (7)	−0.0119 (8)	−0.0038 (8)
O4A	0.0374 (10)	0.0220 (9)	0.0297 (10)	−0.0085 (8)	−0.0177 (8)	0.0095 (7)
N1A	0.0242 (10)	0.0120 (9)	0.0239 (10)	0.0009 (8)	−0.0057 (8)	0.0002 (8)
N2A	0.0254 (11)	0.0149 (10)	0.0227 (11)	−0.0001 (8)	−0.0015 (9)	−0.0022 (8)
C1A	0.0236 (12)	0.0175 (12)	0.0203 (12)	−0.0015 (10)	−0.0004 (10)	0.0016 (10)
C2A	0.0223 (12)	0.0152 (11)	0.0225 (12)	−0.0009 (9)	0.0003 (10)	0.0007 (10)
C3A	0.0189 (11)	0.0165 (11)	0.0188 (12)	0.0010 (9)	−0.0005 (9)	0.0000 (9)
C4A	0.0200 (11)	0.0147 (11)	0.0218 (12)	0.0012 (9)	0.0003 (10)	0.0020 (9)
C5A	0.0199 (11)	0.0156 (11)	0.0203 (12)	−0.0020 (9)	0.0009 (10)	−0.0043 (9)
C6A	0.0225 (12)	0.0201 (12)	0.0197 (12)	0.0009 (10)	−0.0037 (10)	−0.0031 (10)
C7A	0.0385 (14)	0.0200 (13)	0.0315 (14)	−0.0024 (11)	−0.0166 (12)	0.0051 (11)
C8A	0.0212 (12)	0.0177 (12)	0.0200 (12)	0.0010 (9)	−0.0002 (10)	0.0031 (9)
C9A	0.0278 (13)	0.0166 (12)	0.0268 (13)	−0.0029 (10)	−0.0083 (11)	0.0038 (10)
C10A	0.0344 (15)	0.0291 (15)	0.059 (2)	−0.0062 (12)	0.0031 (14)	−0.0046 (14)
C11A	0.0458 (16)	0.0218 (14)	0.0384 (16)	0.0039 (12)	−0.0049 (13)	−0.0032 (12)
C12A	0.0279 (13)	0.0203 (12)	0.0192 (12)	0.0008 (10)	−0.0039 (10)	0.0026 (10)
C13A	0.0455 (17)	0.0416 (17)	0.0267 (14)	−0.0017 (13)	−0.0096 (13)	0.0101 (13)
C14A	0.0418 (16)	0.0252 (14)	0.0318 (15)	−0.0010 (12)	−0.0023 (12)	−0.0050 (11)
C15A	0.0287 (13)	0.0283 (14)	0.0308 (14)	−0.0022 (11)	−0.0075 (11)	0.0021 (11)
SiB	0.0263 (4)	0.0151 (3)	0.0256 (4)	0.0002 (3)	−0.0016 (3)	−0.0021 (3)
O1B	0.0291 (9)	0.0142 (8)	0.0332 (10)	0.0009 (7)	−0.0086 (8)	0.0002 (7)
O2B	0.0364 (10)	0.0172 (9)	0.0363 (10)	−0.0019 (8)	−0.0102 (8)	0.0038 (8)
O3B	0.0268 (9)	0.0247 (9)	0.0334 (10)	0.0016 (7)	−0.0117 (8)	0.0056 (8)
O4B	0.0281 (9)	0.0237 (9)	0.0227 (9)	0.0006 (7)	−0.0085 (7)	−0.0033 (7)
N1B	0.0227 (10)	0.0121 (9)	0.0225 (10)	0.0023 (8)	−0.0049 (8)	0.0002 (8)
N2B	0.0250 (11)	0.0179 (10)	0.0220 (10)	0.0011 (8)	−0.0027 (9)	0.0026 (8)
C1B	0.0237 (12)	0.0167 (12)	0.0220 (12)	0.0002 (10)	0.0006 (10)	−0.0009 (10)
C2B	0.0218 (12)	0.0142 (11)	0.0219 (12)	0.0024 (9)	0.0007 (10)	0.0021 (9)
C3B	0.0191 (11)	0.0175 (12)	0.0206 (12)	0.0009 (9)	−0.0004 (10)	0.0001 (10)
C4B	0.0213 (12)	0.0136 (11)	0.0237 (12)	−0.0005 (9)	0.0015 (10)	−0.0009 (9)
C5B	0.0223 (12)	0.0164 (12)	0.0227 (12)	0.0021 (9)	−0.0006 (10)	0.0022 (10)
C6B	0.0222 (12)	0.0217 (12)	0.0191 (12)	0.0010 (10)	−0.0006 (10)	−0.0001 (10)
C7B	0.0309 (13)	0.0201 (12)	0.0286 (14)	−0.0012 (10)	−0.0095 (11)	−0.0005 (10)
C8B	0.0200 (12)	0.0185 (12)	0.0227 (12)	0.0010 (10)	0.0013 (10)	−0.0031 (10)
C9B	0.0266 (12)	0.0159 (12)	0.0265 (13)	0.0017 (10)	−0.0055 (10)	−0.0018 (10)
C10B	0.0348 (15)	0.0322 (15)	0.0533 (19)	0.0004 (12)	0.0063 (14)	−0.0005 (14)
C11B	0.0473 (17)	0.0226 (14)	0.0481 (18)	0.0031 (12)	−0.0172 (14)	−0.0052 (13)
C12B	0.0353 (14)	0.0226 (13)	0.0215 (13)	0.0013 (11)	−0.0043 (11)	−0.0016 (10)
C13B	0.069 (2)	0.0412 (18)	0.0267 (15)	0.0057 (15)	−0.0072 (14)	−0.0018 (13)
C14B	0.0413 (16)	0.0208 (13)	0.0387 (16)	0.0056 (12)	−0.0108 (13)	0.0004 (12)
C15B	0.0305 (14)	0.0329 (15)	0.0439 (17)	−0.0011 (12)	−0.0106 (12)	0.0007 (13)

### Geometric parameters (Å, °)

**Table:**

SiA—O4A	1.6533 (16)	SiB—O4B	1.6540 (17)
SiA—C11A	1.851 (3)	SiB—C11B	1.852 (3)
SiA—C10A	1.861 (3)	SiB—C10B	1.862 (3)
SiA—C12A	1.879 (2)	SiB—C12B	1.879 (2)
O1A—C8A	1.235 (3)	O1B—C8B	1.230 (3)
O2A—N2A	1.233 (2)	O2B—N2B	1.231 (2)
O3A—N2A	1.228 (2)	O3B—N2B	1.225 (2)
O4A—C7A	1.420 (3)	O4B—C7B	1.414 (3)
N1A—C8A	1.364 (3)	N1B—C8B	1.362 (3)
N1A—C3A	1.409 (3)	N1B—C3B	1.412 (3)
N1A—H1A	0.8600	N1B—H1B	0.8600
N2A—C5A	1.473 (3)	N2B—C5B	1.478 (3)
C1A—C2A	1.387 (3)	C1B—C2B	1.378 (3)
C1A—C6A	1.392 (3)	C1B—C6B	1.396 (3)
C1A—C7A	1.507 (3)	C1B—C7B	1.515 (3)
C2A—C3A	1.398 (3)	C2B—C3B	1.395 (3)
C2A—H2A	0.9300	C2B—H2B	0.9300
C3A—C4A	1.390 (3)	C3B—C4B	1.393 (3)
C4A—C5A	1.375 (3)	C4B—C5B	1.382 (3)
C4A—H4A	0.9300	C4B—H4B	0.9300
C5A—C6A	1.383 (3)	C5B—C6B	1.379 (3)
C6A—H6A	0.9300	C6B—H6B	0.9300
C7A—H7A1	0.9700	C7B—H7B1	0.9700
C7A—H7A2	0.9700	C7B—H7B2	0.9700
C8A—C9A	1.498 (3)	C8B—C9B	1.505 (3)
C9A—H9A1	0.9600	C9B—H9B1	0.9600
C9A—H9A2	0.9600	C9B—H9B2	0.9600
C9A—H9A3	0.9600	C9B—H9B3	0.9600
C10A—H10A	0.9600	C10B—H10D	0.9600
C10A—H10B	0.9600	C10B—H10E	0.9600
C10A—H10C	0.9600	C10B—H10F	0.9600
C11A—H11A	0.9600	C11B—H11D	0.9600
C11A—H11B	0.9600	C11B—H11E	0.9600
C11A—H11C	0.9600	C11B—H11F	0.9600
C12A—C14A	1.534 (3)	C12B—C15B	1.535 (4)
C12A—C13A	1.539 (3)	C12B—C13B	1.537 (4)
C12A—C15A	1.540 (3)	C12B—C14B	1.541 (3)
C13A—H13A	0.9600	C13B—H13D	0.9600
C13A—H13B	0.9600	C13B—H13E	0.9600
C13A—H13C	0.9600	C13B—H13F	0.9600
C14A—H14A	0.9600	C14B—H14D	0.9600
C14A—H14B	0.9600	C14B—H14E	0.9600
C14A—H14C	0.9600	C14B—H14F	0.9600
C15A—H15A	0.9600	C15B—H15D	0.9600
C15A—H15B	0.9600	C15B—H15E	0.9600
C15A—H15C	0.9600	C15B—H15F	0.9600
			
O4A—SiA—C11A	109.65 (11)	O4B—SiB—C11B	109.84 (11)
O4A—SiA—C10A	109.68 (11)	O4B—SiB—C10B	108.94 (12)
C11A—SiA—C10A	109.21 (13)	C11B—SiB—C10B	109.21 (13)
O4A—SiA—C12A	104.02 (9)	O4B—SiB—C12B	104.37 (10)
C11A—SiA—C12A	112.45 (11)	C11B—SiB—C12B	111.97 (12)
C10A—SiA—C12A	111.70 (12)	C10B—SiB—C12B	112.37 (12)
C7A—O4A—SiA	123.43 (15)	C7B—O4B—SiB	123.18 (15)
C8A—N1A—C3A	129.01 (19)	C8B—N1B—C3B	128.91 (19)
C8A—N1A—H1A	115.5	C8B—N1B—H1B	115.5
C3A—N1A—H1A	115.5	C3B—N1B—H1B	115.5
O3A—N2A—O2A	123.41 (19)	O3B—N2B—O2B	123.62 (18)
O3A—N2A—C5A	118.28 (17)	O3B—N2B—C5B	118.09 (19)
O2A—N2A—C5A	118.31 (18)	O2B—N2B—C5B	118.28 (18)
C2A—C1A—C6A	119.3 (2)	C2B—C1B—C6B	119.2 (2)
C2A—C1A—C7A	119.4 (2)	C2B—C1B—C7B	119.2 (2)
C6A—C1A—C7A	121.3 (2)	C6B—C1B—C7B	121.6 (2)
C1A—C2A—C3A	121.7 (2)	C1B—C2B—C3B	122.1 (2)
C1A—C2A—H2A	119.1	C1B—C2B—H2B	118.9
C3A—C2A—H2A	119.1	C3B—C2B—H2B	118.9
C4A—C3A—C2A	119.3 (2)	C4B—C3B—C2B	119.4 (2)
C4A—C3A—N1A	124.29 (19)	C4B—C3B—N1B	124.2 (2)
C2A—C3A—N1A	116.4 (2)	C2B—C3B—N1B	116.38 (19)
C5A—C4A—C3A	117.5 (2)	C5B—C4B—C3B	117.0 (2)
C5A—C4A—H4A	121.3	C5B—C4B—H4B	121.5
C3A—C4A—H4A	121.3	C3B—C4B—H4B	121.5
C4A—C5A—C6A	124.6 (2)	C6B—C5B—C4B	124.7 (2)
C4A—C5A—N2A	118.10 (19)	C6B—C5B—N2B	117.9 (2)
C6A—C5A—N2A	117.30 (19)	C4B—C5B—N2B	117.5 (2)
C5A—C6A—C1A	117.5 (2)	C5B—C6B—C1B	117.5 (2)
C5A—C6A—H6A	121.2	C5B—C6B—H6B	121.2
C1A—C6A—H6A	121.2	C1B—C6B—H6B	121.2
O4A—C7A—C1A	110.39 (19)	O4B—C7B—C1B	112.15 (19)
O4A—C7A—H7A1	109.6	O4B—C7B—H7B1	109.2
C1A—C7A—H7A1	109.6	C1B—C7B—H7B1	109.2
O4A—C7A—H7A2	109.6	O4B—C7B—H7B2	109.2
C1A—C7A—H7A2	109.6	C1B—C7B—H7B2	109.2
H7A1—C7A—H7A2	108.1	H7B1—C7B—H7B2	107.9
O1A—C8A—N1A	122.9 (2)	O1B—C8B—N1B	123.7 (2)
O1A—C8A—C9A	122.75 (19)	O1B—C8B—C9B	122.3 (2)
N1A—C8A—C9A	114.29 (19)	N1B—C8B—C9B	114.01 (19)
C8A—C9A—H9A1	109.5	C8B—C9B—H9B1	109.5
C8A—C9A—H9A2	109.5	C8B—C9B—H9B2	109.5
H9A1—C9A—H9A2	109.5	H9B1—C9B—H9B2	109.5
C8A—C9A—H9A3	109.5	C8B—C9B—H9B3	109.5
H9A1—C9A—H9A3	109.5	H9B1—C9B—H9B3	109.5
H9A2—C9A—H9A3	109.5	H9B2—C9B—H9B3	109.5
SiA—C10A—H10A	109.5	SiB—C10B—H10D	109.5
SiA—C10A—H10B	109.5	SiB—C10B—H10E	109.5
H10A—C10A—H10B	109.5	H10D—C10B—H10E	109.5
SiA—C10A—H10C	109.5	SiB—C10B—H10F	109.5
H10A—C10A—H10C	109.5	H10D—C10B—H10F	109.5
H10B—C10A—H10C	109.5	H10E—C10B—H10F	109.5
SiA—C11A—H11A	109.5	SiB—C11B—H11D	109.5
SiA—C11A—H11B	109.5	SiB—C11B—H11E	109.5
H11A—C11A—H11B	109.5	H11D—C11B—H11E	109.5
SiA—C11A—H11C	109.5	SiB—C11B—H11F	109.5
H11A—C11A—H11C	109.5	H11D—C11B—H11F	109.5
H11B—C11A—H11C	109.5	H11E—C11B—H11F	109.5
C14A—C12A—C13A	109.0 (2)	C15B—C12B—C13B	109.3 (2)
C14A—C12A—C15A	108.7 (2)	C15B—C12B—C14B	108.4 (2)
C13A—C12A—C15A	109.36 (19)	C13B—C12B—C14B	108.6 (2)
C14A—C12A—SiA	110.59 (16)	C15B—C12B—SiB	110.34 (17)
C13A—C12A—SiA	109.21 (17)	C13B—C12B—SiB	110.11 (18)
C15A—C12A—SiA	109.89 (16)	C14B—C12B—SiB	110.04 (17)
C12A—C13A—H13A	109.5	C12B—C13B—H13D	109.5
C12A—C13A—H13B	109.5	C12B—C13B—H13E	109.5
H13A—C13A—H13B	109.5	H13D—C13B—H13E	109.5
C12A—C13A—H13C	109.5	C12B—C13B—H13F	109.5
H13A—C13A—H13C	109.5	H13D—C13B—H13F	109.5
H13B—C13A—H13C	109.5	H13E—C13B—H13F	109.5
C12A—C14A—H14A	109.5	C12B—C14B—H14D	109.5
C12A—C14A—H14B	109.5	C12B—C14B—H14E	109.5
H14A—C14A—H14B	109.5	H14D—C14B—H14E	109.5
C12A—C14A—H14C	109.5	C12B—C14B—H14F	109.5
H14A—C14A—H14C	109.5	H14D—C14B—H14F	109.5
H14B—C14A—H14C	109.5	H14E—C14B—H14F	109.5
C12A—C15A—H15A	109.5	C12B—C15B—H15D	109.5
C12A—C15A—H15B	109.5	C12B—C15B—H15E	109.5
H15A—C15A—H15B	109.5	H15D—C15B—H15E	109.5
C12A—C15A—H15C	109.5	C12B—C15B—H15F	109.5
H15A—C15A—H15C	109.5	H15D—C15B—H15F	109.5
H15B—C15A—H15C	109.5	H15E—C15B—H15F	109.5

### Hydrogen-bond geometry (Å, °)

**Table:**

*D*—H···*A*	*D*—H	H···*A*	*D*···*A*	*D*—H···*A*
N1A—H1A···O1B	0.86	2.13	2.982 (2)	172.
N1B—H1B···O1A^i^	0.86	2.14	2.991 (2)	173.

Symmetry codes: (i) *x*, *y*+1, *z*.
